# Comparative Characterization of the Multiscale Structure, Physicochemical Properties, and In Vitro Digestibility of Starches from Selected Stress-Resistant Rice Varieties

**DOI:** 10.3390/molecules31183255

**Published:** 2026-09-14

**Authors:** Dandan Qin, Lingfeng Zhu, Hongyan Yuan, Xinxin Xia, Zhijun Liu, Menglin Liu, Talha Riaz, Qiutao Xie, Te Yu

**Affiliations:** 1 DongTing Laboratory, Hunan Institute of Agricultural Product Processing and Quality Safety, Hunan Academy of Agricultural Sciences, Changsha 410125, China; qddjgs@hunaas.cn (D.Q.); zlfjgs@hunaas.cn (L.Z.); yhyjgs@hunaas.cn (H.Y.); xiaxinxin@hunaas.cn (X.X.); lml9625@163.com (M.L.); 2 Yuelushan Laboratory, Changsha 410128, China; 3 National Technology Innovation Center for Saline-Alkali Tolerant Rice in Sanya, Sanya 572000, China; liuzhj2026@163.com; 4 National Research and Development Centre for Egg Processing, College of Food Science and Technology, Huazhong Agricultural University, Wuhan 430070, China; talhariaz2844@gmail.com

**Keywords:** stress-resistant rice, rice starch, multiscale structure, physicochemical properties, in vitro digestibility

## Abstract

Stress-resistant rice varieties are valuable for sustainable production and rice-based food development. Six rice varieties selected for salt alkali tolerance or low cadmium accumulation were characterized for starch composition, multiscale structure, physicochemical properties, rheological behavior, and in vitro digestibility. Rice flours contained 78.11% to 83.51% starch, 12.67% to 18.12% amylose, and 5.23% to 7.50% protein. All starches exhibited A-type crystallinity but showed varietal differences in granule morphology, particle size, molecular organization, thermal behavior, pasting properties, freeze–thaw stability, paste clarity, and rheological characteristics. Rapidly digestible starch ranged from 60.44% to 72.79%, slowly digestible starch ranged from 22.40% to 33.75%, and resistant starch ranged from 4.46% to 10.59%. Jingliangyou 3261 exhibited the highest resistant starch content, whereas Xinjiang aromatic rice combined the highest slowly digestible starch content with the lowest rapidly digestible starch content and the highest paste clarity. Ningjing 48 and Xinjiang aromatic rice also showed favorable freeze–thaw stability, highlighting distinct varietal profiles for targeted rice food applications. These findings provide preliminary information for further evaluation of these varieties under practical cooking and food-processing conditions.

## 1. Introduction

As one of the world’s principal cereal crops, rice (*Oryza sativa* L.) plays a crucial role in human nutrition by serving as a major source of dietary energy for a large segment of the global population. Therefore, maintaining stable rice production and grain quality is essential for global food security. However, rice production is increasingly threatened by abiotic stresses, including soil salinity, alkalinity, and heavy-metal contamination [1]. These stresses affect not only crop productivity but also grain quality and food safety. In this context, stress-resistant rice varieties, including salt–alkali-tolerant and low-cadmium-accumulating cultivars, have attracted increasing attention because of their potential value in sustainable rice production and rice-based food development. Beyond agronomic performance, understanding the grain quality and starch characteristics of these varieties is essential for their effective utilization in food systems [2].

Starch constitutes the major fraction of rice grains and is a key determinant of their processing performance, physicochemical properties, and nutritional attributes [3]. Rice starch mainly consists of amylose and amylopectin. The molecular configuration and higher-order organization of these polymers determine starch structures at different hierarchical levels. These structural levels include granule morphology, particle size distribution, crystallinity, short-range molecular order, lamellar organization, and molecular weight characteristics [4]. These features strongly influence functional properties, including water absorption, swelling capacity, solubility, gelatinization behavior, pasting properties, rheological characteristics, and enzymatic digestibility [5]. Furthermore, the digestibility of rice starch, generally categorized as rapidly digestible starch, slowly digestible starch, and resistant starch, depends on starch composition, molecular arrangement, granule integrity, and its interactions with non-starch constituents, including proteins, lipids, and minerals [6]. Therefore, this study aimed to comparatively characterize starch composition, multiscale structure, physicochemical properties, rheological behavior, and in vitro digestibility among selected rice varieties with stress-resistance traits [7].

Previous studies have extensively investigated the relationships between starch structure and functional properties in conventional rice varieties. Park et al. [7] reported that differences in amylose content were associated with variations in the structural, physicochemical, and digestibility characteristics of rice starch, while Liu et al. [8] further demonstrated the important roles of multilevel starch structures in determining the functional properties of high-amylose rice. Zhong et al. [9] systematically revealed relationships among molecular structure, crystalline and lamellar organization, thermal and pasting properties, rheological behavior, and digestibility across different rice genotypes. However, studies on rice carrying stress-resistance traits have predominantly focused on agronomic performance and stress-response mechanisms, whereas their food-quality attributes have received comparatively less attention. For salt-tolerant rice, soil salinity has been shown to alter rice flour composition, starch crystalline and lamellar organization, and related pasting and rheological properties [10]. Research on low-cadmium-accumulating rice has mainly addressed cultivar screening and the physiological mechanisms governing Cd uptake, transport, storage, and redistribution [11,12]. Nevertheless, comparative information integrating starch multiscale structure, physicochemical properties, rheological behavior, and in vitro digestibility within a unified framework remains scarce. The six varieties in the present study were selected to represent two practically important breeding targets for rice production under challenging soil conditions, namely salt-alkali tolerance and low-cadmium accumulation. They were evaluated together to characterize varietal diversity in starch quality among selected rice germplasm with agronomically relevant stress-resistance traits, rather than to compare the two stress-resistance categories as experimental groups. Accordingly, the present study compared six selected rice varieties in terms of chemical composition, starch multiscale structure, physicochemical properties, rheological behavior, and in vitro digestibility. The findings are expected to advance the understanding of varietal differences in starch quality and provide a scientific basis for the targeted utilization of these varieties in rice-based food products.

## 2. Results and Discussion

### 2.1. Basic Chemical Composition of Rice Flour

The basic chemical compositions and trace element contents of rice flour from the six selected rice varieties are shown in Table 1 and Table S1. Significant differences were observed among the six samples in moisture, total starch, amylose, protein, crude fat, ash, and crude fiber contents (*p* < 0.05), indicating clear varietal differences in grain composition. The moisture content ranged from 12.27% to 15.90%, with Zhenliangyou 8612 showing the highest value and Xinjiang aromatic rice showing the lowest. The total starch content ranged from 78.11% to 83.51%, with Jingliangyou 3261 and Xinjiang aromatic rice exhibiting relatively high values. Amylose content varied from 12.67% to 18.12%, with Ningjing 48 showing the highest value, followed by Xinjiang aromatic rice, whereas Jingliangyou 3261 showed the lowest value. Protein content ranged from 5.23% to 7.50%, and Xizi 3 exhibited a significantly higher protein content than the other samples. Trace element analysis showed that Fe, Zn, Mg, Ca, Mn, Cu, Cd, and Se contents also differed among the samples. Fe and Zn contents were highest in Jingliangyou 3261, while Mg and Ca were relatively abundant mineral elements in all samples. The contents of Cd and Se were low, and Cd or Se was not detected in some samples, indicating differences in mineral accumulation among the selected rice varieties. Such differences may arise from varietal genetic background, cultivation conditions, environmental factors, and postharvest processing. Variations in trace element accumulation among rice varieties have also been reported previously. Kodikara et al. [13] demonstrated substantial differences in the elemental profiles of diverse rice varieties and reported relatively low concentrations of toxic trace elements, including Cd. These findings are consistent with the marked varietal dependence of chemical and mineral composition observed in the present study.

The differences in chemical composition may contribute to variations in starch structure and functional properties among the selected rice varieties. Amylose can promote intermolecular association among starch chains and affect swelling, molecular leaching, gel formation, retrogradation, and enzymatic hydrolysis. In this study, Ningjing 48 and Xinjiang aromatic rice showed relatively high amylose contents, whereas Jingliangyou 3261 showed the lowest amylose content, suggesting that amylose may participate in regulating subsequent physicochemical and digestive behaviors. However, the effects of amylose should be interpreted together with other structural factors, including crystalline structure, short-range order, lamellar organization, molecular weight distribution, and non-starch components. Proteins, lipids, and mineral elements may also influence starch hydration, thermal stability, and enzymatic accessibility. Therefore, the differences in grain composition and mineral profiles provide a compositional basis for understanding the varietal differences in starch multiscale structure, physicochemical properties, and digestibility discussed in the following sections [8].

### 2.2. Structural Characteristics of Rice Starch

#### 2.2.1. Morphological Analysis of Rice Flour and Starch

**Figure 1:**
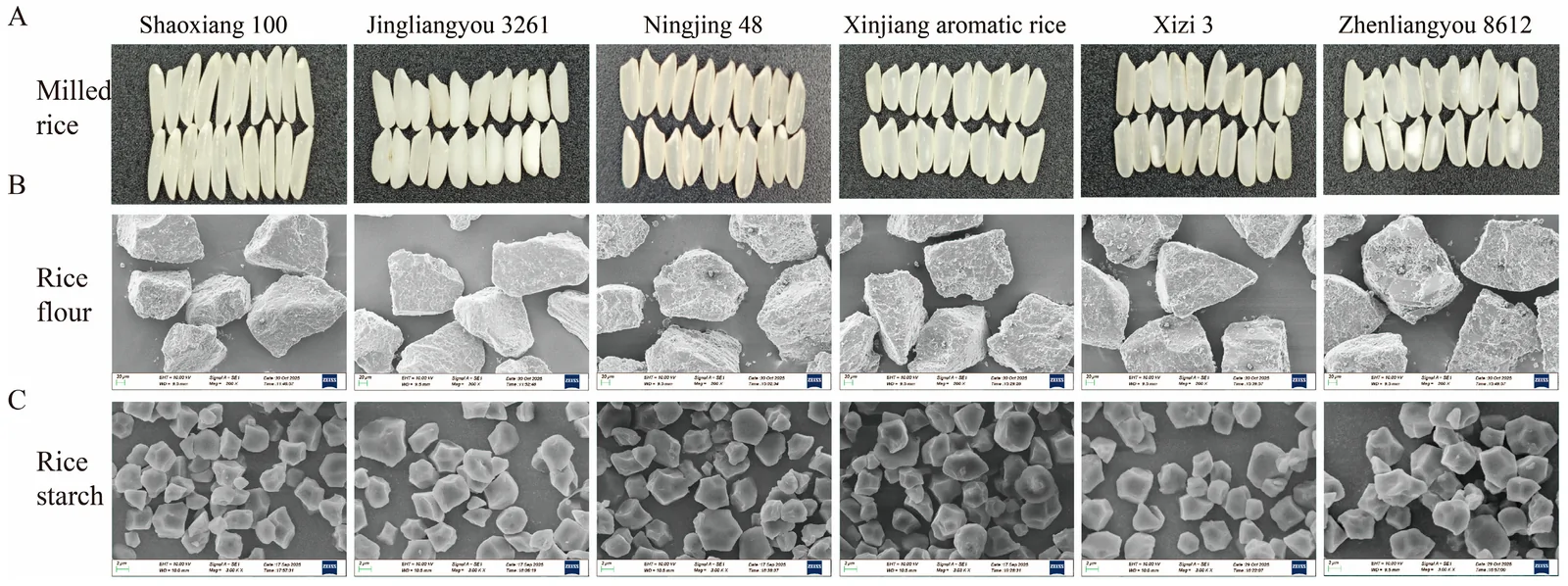
Morphological characteristics of milled rice kernels (**A**), rice flour particles (**B**), and isolated starch granules (**C**) from different stress-resistant rice varieties. (**B**,**C**) show the scanning electron microscope images of rice flour and starch under magnification factors of 200× and 3000×.

The scanning electron microscope images (SEM) of rice flour particles and isolated starch granules from different stress-resistant rice varieties are shown in Figure 1. Rice flour particles from all samples displayed irregular block-like or flake-like structures with rough surfaces, and cracks, depressions, and particle aggregation were observed to varying degrees. These features may be related to the composite structure of rice flour, in which starch granules are embedded or associated with proteins, lipids, and cell wall components [14]. After alkaline extraction, the isolated starch granules showed fewer surface attachments and mainly exhibited polygonal, nearly spherical, or irregular polyhedral shapes with relatively smooth surfaces and clear edges [15]. Nevertheless, differences in granule size and aggregation state were still observed among varieties, indicating varietal differences in starch granule development and endosperm organization. This may suggest differences in moisture content and water absorption capacity among rice varieties. As the moisture content of rice flour increases, small starch granules bind together and aggregate [16].

The morphological differences may further influence starch hydration, gelatinization, and enzymatic hydrolysis. Starch granules with rougher surfaces or partial structural damage generally provide a larger contact area for water and enzymes, whereas granules with smoother surfaces and more intact structures may restrict water penetration and enzyme accessibility. This is also correlated with the texture and mouthfeel of the prepared rice flour [17]. Therefore, the morphological differences of starch particles may be associated with the subsequent changes in physical and chemical properties and in vitro digestibility.

#### 2.2.2. Particle Size Distribution Characteristics

The particle-size parameters of isolated starches from the six rice varieties are summarized in Table 2. D_(10)_, D_(50)_ and D_(90)_ represent the particle sizes corresponding to the cumulative distribution percentages of 10%, 50% and 90%,respectively. D _[3,2]_ represents the Sauter mean diameter, and D _[4,3]_ represents the De Brouckere mean diameter. Significant varietal differences were observed in D_(10)_, D_(50)_, D_(90)_, D _[3,2]_, and D _[4,3]_. Xinjiang aromatic rice exhibited the largest particle-size parameters, with D_(10)_, D_(50)_, and D_(90)_ values of 1.246, 1.376, and 1.526 μm, respectively. In contrast, the corresponding values of the other five varieties were concentrated within relatively narrow ranges of 0.591–0.599, 0.686–0.700, and 0.806–0.817 μm, respectively. Xinjiang aromatic rice also exhibited the highest D _[3,2]_ and D _[4,3]_, demonstrating a distinctly larger particle-size profile than the other varieties.

Granule-size variation is an important structural characteristic of rice starch and is closely associated with its physicochemical and digestive behavior. Previous studies have shown substantial cultivar-dependent differences in rice starch granule size, accompanied by variations in thermal, pasting, and rheological properties [18]. Granule size can also influence enzymatic accessibility by altering the available surface area and the interaction between starch particles and digestive enzymes. However, starch hydrolysis is governed by multiple structural features, including particle size, granule morphology, crystalline organization, and molecular architecture [19]. Accordingly, the distinctly larger particle-size profile of Xinjiang aromatic rice represents a characteristic granule-level structural feature that, together with its molecular and ordered structural characteristics, may contribute to its differentiated physicochemical and in vitro digestive behavior observed in subsequent analyses.

Differences in particle size distribution may be associated with varietal genetic background, endosperm development, and starch biosynthesis. Smaller particles generally have a larger specific surface area and may be more readily hydrated and attacked by enzymes, whereas larger particles may possess greater structural integrity and lower surface activity. Thus, the larger particle size of Xinjiang aromatic rice and the narrower distribution of Ningjing 48 may contribute to their distinct swelling, gelatinization, rheological, and digestive behaviors in subsequent analyses.

#### 2.2.3. Analysis of Starch Crystalline Structure by X-Ray Diffraction (XRD)

**Figure 2:**
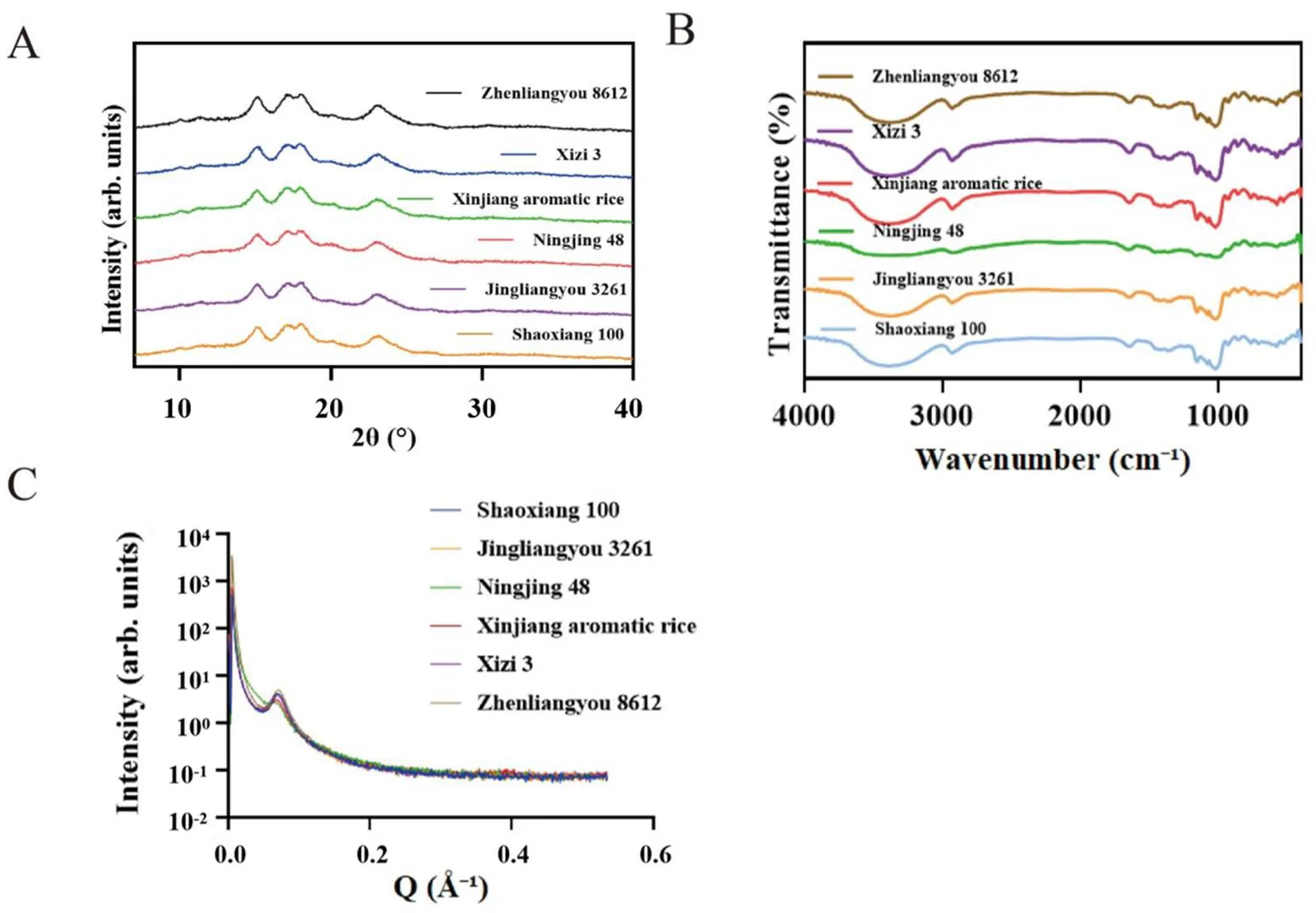
Crystalline, short-range ordered, and lamellar structures of starches from six stress-resistant rice varieties. (**A**) X-ray diffraction patterns; (**B**) Fourier transform infrared spectra; (**C**) Small-angle x-ray scattering profiles.

The XRD patterns of isolated starches from different stress-resistant rice varieties are shown in Figure 2A. All starch samples exhibited characteristic diffraction peaks at approximately 2θ = 15°, 17°, 18°, and 23°, with a typical doublet peak at 17–18°, indicating that all starches possessed a typical A-type crystalline structure. This result suggests that the crystalline polymorph of rice starch was similar among the selected varieties, although the degree of crystalline order differed. The relative crystallinity varied among the samples, with Jingliangyou 3261 showing the highest value of 20.27%, followed by Zhenliangyou 8612 at 19.39%. The relative crystallinities of Shaoxiang 100, Xizi 3, Xinjiang aromatic rice, and Ningjing 48 were 18.72%, 18.55%, 18.10%, and 17.38%, respectively. The differences in relative crystallinity reflect varietal variation in the organization of ordered starch domains. Crystalline regions of starch are mainly associated with the ordered packing of amylopectin double helices, and their stability can influence hydration, gelatinization, and enzymatic accessibility [20]. Notably, Jingliangyou 3261 exhibited the highest relative crystallinity, representing a distinctive combination of crystalline organization among the six starches.

#### 2.2.4. Analysis of Starch Ordered Structure by Fourier Transform Infrared Spectroscopy (FTIR)

The FTIR spectra of the six rice starches showed similar characteristic absorption bands, indicating no marked differences in their major chemical functional groups (Figure 2B). The absorption bands near 1047, 1022, and 995 cm^−1^ are sensitive to changes in the local molecular organization of starch. The 1047/1022 cm^−1^ and 1022/995 cm^−1^ ratios are widely used as an indicator to measure the crystallinity and molecular order of starch [21]. Clear varietal differences were observed in these spectral indices. Ningjing 48 exhibited the highest 1047/1022 cm^−1^ ratio, followed by Jingliangyou 3261 and Shaoxiang 100, indicating more pronounced short-range molecular ordering in these starches. In contrast, Xinjiang aromatic rice, Xizi 3, and Zhenliangyou 8612 showed lower 1047/1022 cm^−1^ ratios, reflecting distinct local molecular arrangements. The 1022/995 cm^−1^ ratio was highest in Xinjiang aromatic rice and lowest in Ningjing 48, further demonstrating varietal differentiation in the local starch chain environment.

These FTIR results provide structural information beyond the crystalline polymorph identified by XRD. Although all six starches exhibited an A-type crystalline pattern, their FTIR spectral indices differed markedly, demonstrating that starches with the same crystalline polymorph can retain distinct short-range molecular arrangements. In particular, Ningjing 48 was characterized by a more pronounced short-range ordered structure, whereas Xinjiang aromatic rice exhibited a contrasting local molecular organization. These differences further highlight the multiscale structural diversity of starch among the six rice varieties.

#### 2.2.5. Small-Angle X-Ray Scattering (SAXS) Analysis of Rice Starch

The SAXS profiles of isolated starches from the six selected rice varieties are shown in Figure 2C, and the quantitative SAXS parameters are summarized in Table 3. All starches exhibited a characteristic scattering peak within a similar q region, indicating a broadly conserved semicrystalline lamellar periodicity among the six rice starches. The corresponding lamellar repeat distance (d), calculated according to d = 2π/q_peak, showed varietal variation, reflecting differences in the periodic spacing of the alternating crystalline and amorphous lamellae. SAXS has been widely used to characterize the repeat architecture of semicrystalline starch lamellae, and the position of the characteristic scattering peak provides a quantitative basis for determining lamellar periodicity [22].

The differences in d among the rice starches indicate variation in lamellar repeat architecture rather than differences in lamellar ordering degree. Previous research on rice starch has demonstrated that lamellar structural parameters are associated with molecular composition and crystalline organization, and that changes in the relative organization of crystalline and amorphous regions can be accompanied by differences in lamellar repeat distance [9]. Thus, the present SAXS results provide an additional structural dimension for distinguishing the six rice starches beyond their crystalline polymorphism and short-range molecular organization.

When considered together, the XRD, FTIR, and SAXS results reveal structural differentiation at complementary hierarchical levels. XRD showed that all six starches retained an A-type crystalline polymorph but differed in relative crystallinity, whereas FTIR revealed varietal differences in short-range molecular organization. SAXS further demonstrated differences in semicrystalline lamellar periodicity. Therefore, the starches shared a broadly similar crystalline type while retaining distinct organization at the crystalline, local molecular, and lamellar levels. Previous studies have also shown that crystalline, lamellar, and granular structural features can jointly influence starch digestion behavior rather than acting independently [20]. Accordingly, the lamellar differences observed here, together with the molecular and crystalline characteristics, may contribute to the varietal differences in physicochemical properties and in vitro digestibility described in the subsequent sections.

#### 2.2.6. Gel Permeation Chromatography (GPC) Analysis

The apparent molecular weight distribution of starches from different stress-resistant rice varieties is shown in Table 4. Clear varietal differences were observed in molecular weight parameters, indicating differences in starch molecular chain composition and distribution. The number-average molecular weight (Mn) ranged from 3.04 × 10^5^ to 4.18 × 10^5^, while the weight-average molecular weight (Mw) ranged from 4.93 × 10^6^ to 1.28 × 10^7^. Xinjiang aromatic rice showed the highest Mw value, followed by Zhenliangyou 8612, Xizi 3, and Ningjing 48, whereas Shaoxiang 100 showed the lowest Mw value. The polydispersity index (PDI) ranged from 16.22 to 32.82, with Xinjiang aromatic rice showing the broadest molecular weight distribution and Shaoxiang 100 showing the narrowest distribution. In addition, Xizi 3 exhibited the highest apparent Mz, indicating a greater contribution of high-molecular-weight fractions to its distribution profile. These apparent molecular-weight distributions clearly differentiated the six rice starches at the molecular scale. Xinjiang aromatic rice was distinguished by the highest apparent Mw and the broadest molecular-weight distribution, whereas Shaoxiang 100 exhibited the lowest apparent Mw and the narrowest distribution, demonstrating pronounced varietal differences in molecular-size distribution.

### 2.3. Analysis of Physical and Chemical Properties of Rice Starch

#### 2.3.1. Swelling Power and Solubility of Starch

The solubility and swelling power of starches from the six selected rice varieties at 50, 70, and 90 °C are shown in Table S2. Both parameters increased with increasing temperature, although the magnitude of the increase differed among varieties. At 50 °C, all starches exhibited relatively low solubility and swelling power, consistent with limited hydration and molecular leaching below the major gelatinization transition. At 70 °C, both parameters increased markedly, with Ningjing 48 and Xinjiang aromatic rice showing comparatively pronounced hydration and swelling responses. At 90 °C, solubility increased further, and Zhenliangyou 8612, Xinjiang aromatic rice, and Ningjing 48 exhibited relatively high values, whereas Jingliangyou 3261 maintained a comparatively low solubility. This finding is consistent with the results reported by Chau et al. [23]. These results demonstrate clear varietal differentiation in starch hydration, swelling, and molecular leaching during heating.

Swelling power and solubility represent related but distinct responses of starch granules to heating in excess water. Swelling power primarily reflects water uptake and expansion of starch granules, whereas solubility is associated with the release of starch components into the aqueous phase. Previous studies on rice starch have shown that these properties are associated with multiple structural characteristics, including amylose content, molecular architecture, relative crystallinity, short-range order, and lamellar structure, rather than being controlled by a single structural parameter [24,25,26]. In the present study, the swelling and solubility patterns can therefore be considered together with the independently measured differences in amylose content, relative crystallinity, FTIR-derived short-range molecular order, SAXS-derived lamellar periodicity, and apparent molecular-weight distribution. Notably, Ningjing 48 and Xinjiang aromatic rice exhibited relatively high amylose contents but also pronounced swelling responses at 70 °C, indicating that amylose content alone does not account for the observed hydration behavior. Instead, the variety-specific swelling and solubility profiles are consistent with the combined contribution of starch composition and multiscale structural organization. These differentiated hydration characteristics provide a functional basis for the subsequent varietal differences in gelatinization, pasting, and rheological behavior.

#### 2.3.2. Analysis of the Freeze-Thaw Stability of Rice Starch

**Figure 3:**
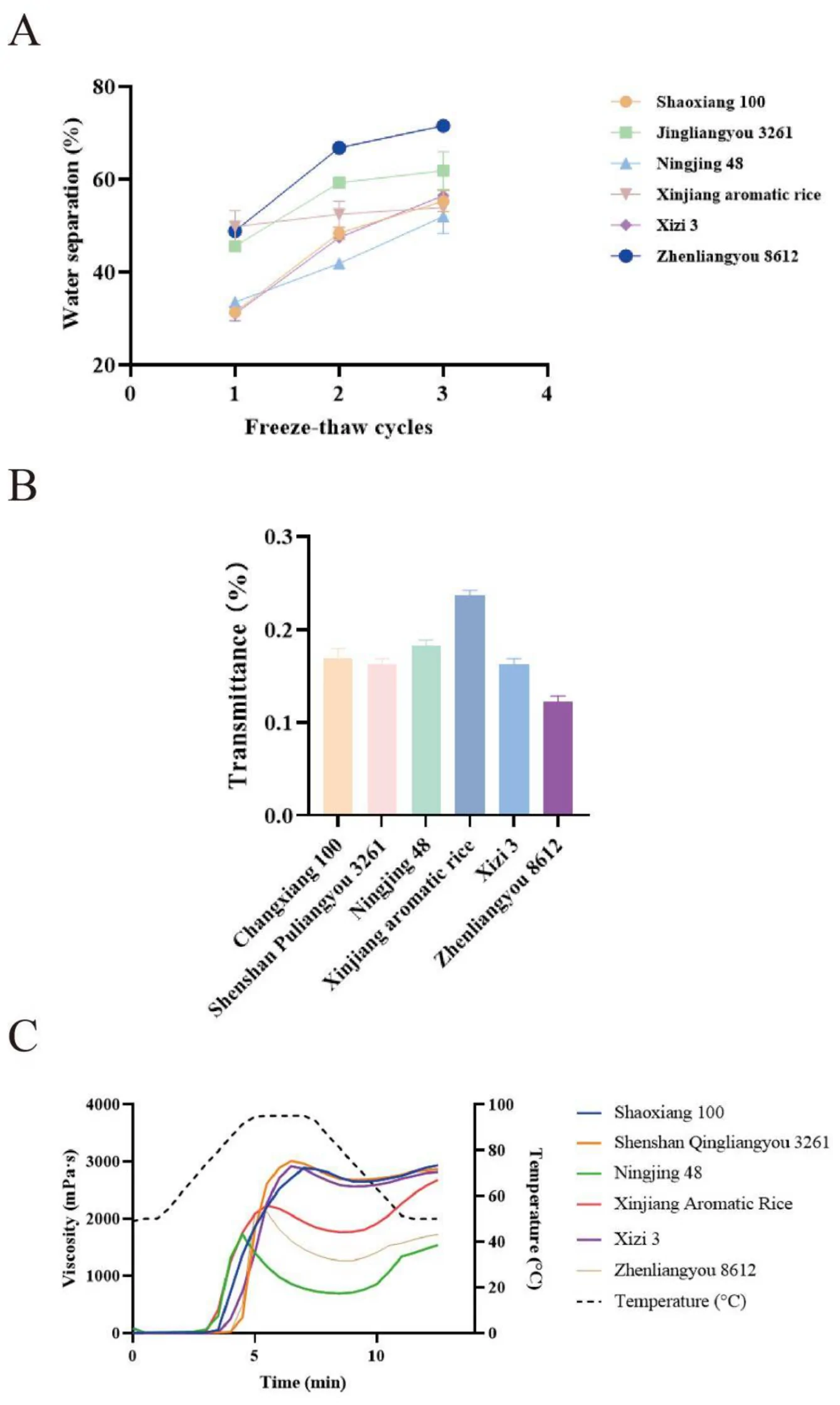
Freeze–thaw stability, paste transparency, and pasting behavior of starches from six stress-resistant rice varieties. (**A**) Freeze–thaw syneresis of starch gels after one, two, and three freeze–thaw cycles; (**B**) light transmittance of starch pastes; (**C**) pasting viscosity profiles determined by rapid visco analysis.

The freeze–thaw syneresis rates of starch gels from different stress-resistant rice varieties are shown in Figure 3A. As the number of freeze–thaw cycles increased from one to three, the syneresis rates of all samples generally increased, indicating that repeated freezing and thawing reduced the water-holding capacity of starch gel systems and promoted the release of free or weakly bound water. After the first freeze–thaw cycle, Xinjiang aromatic rice, Zhenliangyou 8612, and Jingliangyou 3261 showed relatively high syneresis rates of 49.76 ± 3.47%, 48.96 ± 1.06%, and 45.56 ± 1.07%, respectively, whereas Xizi 3, Shaoxiang 100, and Ningjing 48 showed lower values of 30.97 ± 1.53%, 31.32 ± 0.98%, and 33.46 ± 0.68%, respectively. After three freeze–thaw cycles, Zhenliangyou 8612 exhibited the highest syneresis rate, reaching 71.24 ± 0.87%, indicating the weakest freeze–thaw stability, while Xinjiang aromatic rice and Ningjing 48 showed relatively lower values of 53.62 ± 0.93% and 51.99 ± 3.68%, respectively. Water separation during freeze–thaw treatment is mainly associated with ice crystal formation, starch chain rearrangement, retrogradation, and contraction of the gel network [27]. Therefore, the differences in syneresis among varieties suggest that the starch gels differed in molecular reassociation behavior and water retention ability during low-temperature cycling.

Ningjing 48 and Xinjiang aromatic rice retained comparatively better freeze—thaw stability after repeated cycles, whereas Zhenliangyou 8612 showed a greater tendency toward syneresis. This behavior makes Ningjing 48 and Xinjiang aromatic rice potentially more suitable for starch-based foods exposed to repeated freezing and thawing. Further evaluation in formulated food systems would be needed to confirm this advantage under practical processing conditions.

#### 2.3.3. Light Transmittance of Starch Paste

The light transmittance of starch pastes from the six selected rice varieties is shown in Figure 3B. Significant differences were observed among the six samples, demonstrating clear varietal differences in paste transparency. Xinjiang aromatic rice exhibited the highest transmittance, approximately 0.24, followed by Ningjing 48 at approximately 0.18. Shaoxiang 100, Xizi 3, and Jingliangyou 3261 showed intermediate values of approximately 0.16–0.17, whereas Zhenliangyou 8612 exhibited the lowest transmittance, approximately 0.12. Thus, Xinjiang aromatic rice was distinguished by having the highest paste clarity among the six starches.

Light transmittance provides an optical measure of starch paste clarity, and previous studies have shown that paste transparency varies with starch source, composition, and physicochemical characteristics. In the present study, the differences in paste transparency occurred together with independently measured varietal differences in amylose content, swelling behavior, relative crystallinity, short-range molecular order, lamellar periodicity, and apparent molecular-weight distribution. Notably, Xinjiang aromatic rice combined the highest light transmittance with a relatively high amylose content, pronounced swelling behavior at 70 °C, and a distinctive molecular-size distribution, indicating that paste clarity cannot be explained by amylose content or any other single structural parameter alone. Rather, the observed transparency profiles are consistent with the combined contribution of starch composition and multiscale structural organization. The comparatively high paste clarity of Xinjiang aromatic rice therefore represents a distinctive functional characteristic that may be advantageous for starch-based products in which visual clarity is desirable.

#### 2.3.4. Thermal Properties of Starch Determined by Differential Scanning Calorimetry (DSC)

The thermal properties of starches from different stress-resistant rice varieties are shown in Table S3. Significant differences were observed in the gelatinization parameters among the samples, indicating varietal differences in thermal transition behavior. The onset temperature (To), peak temperature (Tp), and conclusion temperature (Tc) ranged from 59.90 to 75.60 °C, 66.38 to 81.60 °C, and 75.57 to 87.33 °C, respectively. Jingliangyou 3261 and Zhenliangyou 8612 exhibited relatively high gelatinization temperatures, whereas Xinjiang aromatic rice and Ningjing 48 showed lower transition temperatures. A significant reduction in To or even Tp implies the formation of abundant new, less stable amylopectin crystals during storage [28].

The gelatinization enthalpy (ΔH) ranged from 8.65 to 14.93 J/g, with Zhenliangyou 8612 showing the highest value and Xinjiang aromatic rice showing the lowest. These differences reflect variation in the temperature range and energy requirement associated with starch gelatinization. A higher ΔH generally indicates that more energy is required to disrupt ordered double-helical structures during gelatinization, whereas a lower ΔH reflects a lower enthalpic requirement for this transition. Previous studies have similarly reported that rice starch gelatinization characteristics are influenced by amylose content, amylopectin molecular structure, crystalline organization, and lamellar architecture [29]. Therefore, the starches from the selected stress-resistant rice varieties differed markedly in thermal transition behavior, which may be associated with differences in crystallinity, short-range order, lamellar organization, and molecular chain interactions.

#### 2.3.5. Pasting Properties Determined by Rapid Visco Analysis (RVA)

The pasting viscosity profiles of starches from different stress-resistant rice varieties are shown in Figure 3C. During heating, the viscosity of all samples increased rapidly after approximately 4–6 min. This increase is consistent with progressive starch gelatinization and the development of a swollen granule-rich paste structure. Most samples reached a peak viscosity and subsequently showed varying degrees of viscosity reduction during the high-temperature holding stage, which may reflect reduced structural integrity of the swollen starch granules under combined thermal and shear treatment. During cooling, viscosity increased again in some samples, a behavior commonly associated with molecular reassociation and the development of a more structured paste network.

Clear varietal differences were observed in the pasting profiles. Shaoxiang 100, Jingliangyou 3261, and Xizi 3 showed relatively high peak viscosities, reflecting a greater capacity to develop viscosity during heating. Ningjing 48 exhibited a lower peak viscosity and a pronounced decrease after reaching the peak, indicating comparatively lower paste stability under the applied heating and shear conditions. Xinjiang aromatic rice showed a more moderate viscosity response, whereas Zhenliangyou 8612 displayed intermediate peak and final viscosities. These differences in RVA behavior may be associated with variation in starch composition, molecular organization, crystalline structure, and interactions developed during gelatinization and cooling. The results therefore demonstrate clear varietal differences in pasting behavior, while the underlying molecular events should be regarded as plausible interpretations rather than direct observations from RVA measurements.

#### 2.3.6. Rheological Properties of Starch from Stress-Resistant Rice Varieties

**Figure 4:**
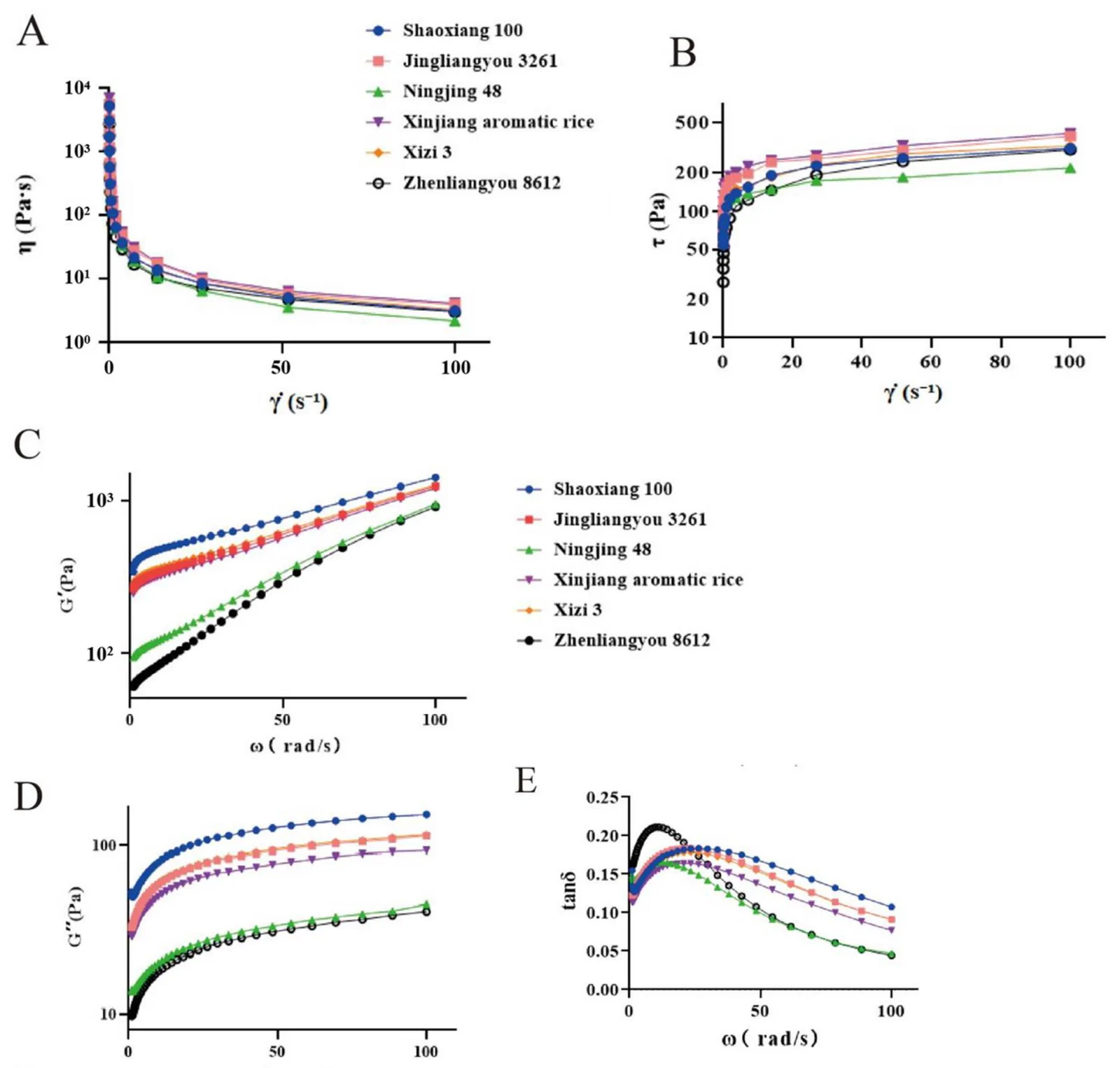
Rheological properties of starch pastes from six stress-resistant rice varieties. (**A**) Apparent viscosity as a function of shear rate; (**B**) shear stress as a function of shear rate; (**C**) storage modulus (G′); (**D**) loss modulus (G″); (**E**) loss tangent (tan δ) as a function of angular frequency.

The steady shear rheological behavior of starch pastes obtained from different stress-resistant rice varieties is illustrated in Figure 4A,B. The apparent viscosity of all starch pastes gradually declined as the shear rate increased, demonstrating typical shear-thinning characteristics. This phenomenon suggests that the internal network structure and molecular chain entanglement of starch pastes were gradually disrupted under increasing shear force, resulting in improved flowability. Clear varietal differences were observed in the apparent viscosity curves. Ningjing 48 showed relatively low apparent viscosity and shear stress, indicating weaker paste structure and lower resistance to flow, whereas the other samples exhibited relatively similar viscosity and shear stress profiles. These differences may be associated with variations in granule swelling, amylose leaching, molecular chain entanglement, and molecular weight distribution after gelatinization. Overall, the steady shear results indicate that starch pastes from different stress-resistant rice varieties differed in flow behavior and structural strength, which may influence their processing stability and texture formation in starch-based food systems.

The dynamic rheological characteristics of the starch pastes are presented in Figure 4C–E. Both the storage modulus (G′) and loss modulus (G″) increased with increasing angular frequency, indicating frequency-dependent viscoelastic behavior. For all samples, G′ remained higher than G″ throughout the tested frequency range, and tan δ values were below 1, showing that the starch pastes exhibited predominantly elastic and weak-gel characteristics. Differences in G′, G″, and tan δ among the varieties further reflected variations in gel strength and viscoelastic balance. Increases in G′ and G″ indicate a harder texture of rice starches [30]. These rheological differences may be associated with varietal differences in starch composition, molecular organization, and the extent of molecular reassociation during gelatinization and cooling.

### 2.4. In Vitro Digestibility of Starch from Stress-Resistant Rice Varieties

The in vitro digestibility profiles of gelatinized and freeze-dried starch samples from different stress-resistant rice varieties are presented in Table 5. Significant variations in rapidly digestible starch (RDS), slowly digestible starch (SDS), and resistant starch (RS) contents were detected among the six varieties (*p* < 0.05), indicating pronounced varietal differences in digestive capabilities. The RDS content ranged from 60.44% to 72.79%. Xizi 3 and Ningjing 48 showed the highest RDS contents at 72.79% and 72.74%, respectively, suggesting faster enzymatic hydrolysis. In contrast, Xinjiang aromatic rice and Jingliangyou 3261 showed the lowest RDS contents at 60.44% and 60.54%, respectively. The SDS content ranged from 22.40% to 33.75%, with Xinjiang aromatic rice showing the highest value, followed by Shaoxiang 100. The RS content ranged from 4.46% to 10.59%, with Jingliangyou 3261 showing the highest value, followed by Zhenliangyou 8612. This characteristic makes Jingliangyou 3261 and Zhenliangyou 8612 potentially more suitable for digestive-resistant starch-based foods.

The differences in digestibility appear to arise from the combined effects of molecular characteristics, ordered structures, and physicochemical behavior rather than from a single structural parameter. Xinjiang aromatic rice exhibited the lowest RDS content and the highest SDS content and was also characterized by the highest apparent Mw and the broadest molecular weight distribution. These molecular characteristics coincided with a digestibility profile characterized by the lowest RDS and highest SDS contents. Jingliangyou 3261, in contrast, exhibited the highest RS content together with the highest relative crystallinity, suggesting that its ordered structural characteristics may contribute to greater resistance to enzymatic hydrolysis. Nevertheless, the correlation analysis showed that RS was not strongly correlated with most individual compositional or structural parameters, indicating that resistance to digestion cannot be attributed to crystallinity or any other single factor alone. Previous studies have similarly shown that rice starch digestibility is jointly influenced by amylose and amylopectin molecular characteristics, crystalline organization, and multilevel structural features [7,8,9].

### 2.5. Correlation Analysis

Pearson correlation analysis was used as an exploratory approach to examine association patterns among chemical composition, molecular characteristics, thermal properties, and in vitro digestibility across the six rice varieties (*n* = 6) (Figure S1). Moisture content showed positive associations with the gelatinization parameters To, Tp, Tc, and ΔH, whereas amylose content showed negative associations with To and Tp. Total starch content was positively associated with Mn, while Mw, Mv, and PDI showed coordinated positive associations with one another, reflecting varietal differences in molecular-size distribution. These relationships demonstrate that variation in starch composition and molecular organization was accompanied by differentiated thermal behavior. Previous studies have similarly shown that amylose content, amylopectin fine structure, and the organization of crystalline and lamellar domains jointly influence the gelatinization behavior of rice starch [31].

Studies have shown that starch digestion behavior reflects the combined contribution of molecular, crystalline, lamellar, and granular structural features rather than a single structural parameter [32]. The correlation analysis revealed that the digestive properties were not closely associated with any single measured composition or structural feature. This might be because the starch samples were gelatinized and freeze-dried before the digestion assay, whereas SEM, XRD, FTIR, and SAXS primarily characterized the native starch structures. Therefore, the observed digestion behavior reflects not only native granular and ordered structures but also structural disruption and subsequent molecular reassociation occurring during sample preparation. This may partly explain the absence of strong correlations between digestion behavior and individual native structural parameters.

## 3. Materials and Methods

### 3.1. Experimental Materials

Six selected rice varieties with stress-resistance traits were used in this study, including three salt–alkali-tolerant varieties, namely Jingliangyou 3261, Ningjing 48, and Xinjiang aromatic rice, and three low-cadmium-accumulating varieties, namely Shaoxiang 100, Xizi 3, and Zhenliangyou 8612. Shaoxiang 100, Jingliangyou 3261, Xizi 3, and Zhenliangyou 8612 are indica rice varieties, whereas Ningjing 48 and Xinjiang aromatic rice are japonica rice varieties. All rice varieties were provided by the National Center of Technology Innovation for Saline-Alkali Tolerant Rice and harvested in the same season. For each variety, one grain batch was collected and processed under identical milling conditions. Starch isolated from each batch was used for subsequent structural, physicochemical, rheological, and digestibility analyses. According to the test results, cadmium was not detected in Shaoxiang 100, Ningjing 48, or Xinjiang aromatic rice, whereas the cadmium contents of Jingliangyou 3261, Xizi 3, and Zhenliangyou 8612 were 0.00327, 0.02870, and 0.04010 mg/kg, respectively. These values were below the maximum limit for cadmium in rice specified in the Chinese national food safety standard.

### 3.2. Preparation of Rice Flour and Starch

The grains were first dehydrated to about 12% moisture, milled into powder, passed through a 100-mesh sieve (0.15 mm) to obtain fine flour, which was then kept in sealed bags away from light before analysis.

Starch extraction was carried out multiple times for each batch of rice samples. Starch extraction was carried out according to the following steps. The rice flour was accurately weighed into a beaker, and n-hexane was added at a 1:5 (g/mL) ratio. The beaker was placed in an electrically heated constant-temperature water bath (DZKW-S-6, Beijing Yongguang Medical Instrument Co.; Ltd.; Beijing, China) and magnetically stirred for 2 h. After that, the mixture was allowed to stand, and the n-hexane was discarded. A 0.4% sodium hydroxide solution was added at a 1:9 (g/mL) ratio. The mixture was magnetically stirred for 3 h and then centrifuged at 1800 *g* for 15 min. The supernatant was discarded, and the precipitate was washed with ultrapure water and centrifuged at 1800 g for 15 min. This washing step was repeated three times. The precipitate was soaked in 0.2% hydrochloric acid for 30 min to adjust its pH to 7.0. The precipitate was then washed with ethanol and centrifuged at 1800 *g* for 15 min. Finally, the precipitate was dried in an oven at 40 °C to obtain the starch. After drying, the starch was crushed, passed through a 100-mesh sieve (0.15 mm), collected, and stored in a dry and dark environment until subsequent use.

### 3.3. Chemical Composition of Rice Flour

The chemical composition of rice flour was evaluated in triplicate. Moisture was determined by drying at 105 °C to constant weight (DHG-9140Aelectric blast drying oven, Beijing Hengtaifeng, Beijing, China), whereas total starch was quantified enzymatically after gelatinization and hydrolysis to glucose. Amylose, protein, crude fat, ash, and crude fiber were analyzed following Nawaz et al. [14] with minor modifications. Amylose was assayed by iodine colorimetry at 720 nm using a UV–visible spectrophotometer (A360, Aoe Instruments, Shanghai, China). Protein was estimated by the Kjeldahl method with a nitrogen conversion factor of 5.95 (HGK-50 Kjeldahl Nitrogen Analyzer, Heguan, China), and crude fat was extracted with petroleum ether in a Soxhlet apparatus. Ash was obtained after muffle furnace (BF51894JC-1, Lindberg/Blue M, Waltham, MA, USA) incineration, while crude fiber was assessed through sequential acid–alkali digestion. Unless otherwise specified, data were reported on a dry-weight basis.

### 3.4. Trace Element Analysis

Fe, Zn, Mg, Ca, Mn, Cu, Cd, and Se were analyzed using an ICP–MS system equipped with an Agilent 7800 mass spectrometer (Agilent Technologies, Santa Clara, CA, USA). The specific operation steps were as follows. Rice flour (0.2 g) was weighed and placed into a microwave digestion instrument (Aurora 6, PreeKem, Shanghai, China). Then, 5–10 mL of nitric acid was added, and the container was covered and left for 1 h. The lid was tightened, and digestion was carried out according to the standard operating procedures of the microwave digestion instrument. After cooling, the container was removed, and the lid was slowly opened to release the gas. The inner lid was rinsed with a small amount of water. The digestion container was then placed on a temperature-controlled electric heating plate heated at 100 °C for 30 min. The volume was made up to 20 mL with water, mixed well, and stored for later use. At the same time, a blank test was performed. The results were calculated according to the following formula:

(1) $$X=\frac{(\rho -\rho_{0})\times V\times f}{m\times 1000}$$

where *X* represents the content of the analyte in the sample, (mg/kg); ρ represents the elemental measurement value of the sample digestion solution, (g/L); $\rho_{0}$ represents the elemental measurement value of the sample blank, (g/L); *V* represents the volume of the sample digestion solution for dilution, (mL); *m* represents the sample mass, (g); *f* represents the dilution factor of the sample; and 1000 represents the unit conversion.

### 3.5. Digital Imaging of Milled Rice Kernels

Intact milled rice grains were selected after removing broken, damaged, discolored grains, and visible impurities. For each variety, rice grains were randomly arranged on a dark background without overlap, and images were captured using the rear camera of a Xiaomi 15 Pro smartphone (Xiaomi, Beijing, China) under fixed lighting, shooting distance, and angle conditions. The obtained images were used to compare the appearance of milled rice kernels among the six selected rice varieties [33].

### 3.6. Multiscale Structural Characterization of Rice Starch

#### 3.6.1. SEM

Rice flour particle morphology and isolated starch granule structure were observed using SEM (EVOLS10, ZEISS, Oberkochen, Germany). Dried samples were mounted on aluminum stubs with double-sided conductive tape, gently cleared of excess powder, and sputter-coated with platinum to improve conductivity. The image acquisition parameters were: an acceleration voltage of 10 kV, and 200× or 3000× magnification [34].

#### 3.6.2. Particle Size Distribution by Laser Diffraction

The particle size distribution of isolated rice starch was determined by laser diffraction using a Mastersizer 2000 laser particle size analyzer (Malvern, Malvern, UK). Starch samples were dispersed in distilled water at a concentration of 1 g/100 mL and continuously stirred until a homogeneous suspension was obtained. Measurements were performed at 25 °C in triplicate. The refractive indices of the starch particles and the dispersant were set at 1.480 and 1.330, respectively. The particle size distribution was characterized by D_(10)_, D_(50)_, D_(90)_, the Sauter mean diameter D _[3,2]_, and the De Brouckere mean diameter D _[4,3]_.

#### 3.6.3. X-Ray Diffraction Analysis

Diffraction data of isolated starch were collected using X-ray diffractometer equipped with Cu/Kα radiation (D8, Bruker, Karlsruhe, Germany). The starch powders were mounted in the sample holder and scanned within the 2θ range of 4–40° under 40 kV and 30 mA. The scanning speed and step size were 2°/min and 0.05°, respectively. The crystalline pattern was identified from the diffraction profiles, and relative crystallinity (RC) was calculated with Jade 6.5 software by integrating the crystalline peak area and total diffraction area according to the following equation:

(2) $$\mathrm{RC}(\%)\;=\;\frac{A_{c}}{A_{a}+A_{c}}\times 100\%$$

where A_a_ represents the area of non-crystalline region, and A_c_ represents the area of the crystalline region.

#### 3.6.4. FTIR Analysis

The short-range ordered structure of the isolated rice starch was analyzed using an FTIR spectrometer (Nicolet 470, Thermo Fisher Scientific, Waltham, MA, USA), following the method of Duan et al. [35] with minor modifications. Dried starch and anhydrous KBr were combined at a ratio of 1:100, fully ground, and pressed into clear tablets for spectral acquisition. Spectra were obtained within the 4000–400 cm^−1^ range at 4 cm^−1^ resolution after 32 scans per sample.

#### 3.6.5. SAXS Analysis

The lamellar organization of rice starch was analyzed by SAXS according to Liu et al. [36] with slight modifications. Briefly, starch was mixed with deionized water to prepare a hydrated starch suspension and equilibrated overnight at room temperature before analysis. SAXS measurements were performed using a SAXSpoint 2.0 instrument (Anton Paar, Graz, Austria) equipped with an Eiger 1M detector. The instrument was operated at 50 kV and 1 mA using X-rays with a wavelength of 0.15418 nm. The sample thickness and sample-to-detector distance were 1 mm and 542 mm, respectively, and each sample was measured twice with an exposure time of 20 min per scan. Deionized water was measured as the blank for background correction. The scattering data were expressed as scattering intensity, I(q), as a function of the scattering vector q, over a q range of approximately 0.00043–0.53449 Å^−1^. The characteristic scattering peak position (q_peak) associated with the semicrystalline lamellar periodicity was determined from the I(q) profile. The corresponding lamellar repeat distance (d) was calculated according to Equation (3):

d = 2π/q_peak(3)

where q_peak is the scattering vector at the characteristic SAXS peak and d represents the repeat distance of the alternating crystalline and amorphous lamellae. The q_peak and d values were used as quantitative parameters to compare lamellar periodicity among the six rice starches, whereas differences in absolute peak intensity were not used as direct indicators of lamellar ordering.

#### 3.6.6. GPC Analysis

The starch sample (4 mg) was weighed and dissolved in 2 mL of DMSO. The solution was stirred until it became completely clear and no visible insoluble substances remained. The resulting solution was filtered through a 0.45 μm organic membrane filter prior to analysis. The injection volume was 100 μL. The relative molecular weight distribution of the starch was determined by gel permeation chromatography using an Agilent 1100 liquid chromatograph (Agilent, Santa Clara, CA, USA). The GPC system was equipped with a differential refractive index detector and a PL gel Mixed-B column (7.5 × 300 mm). DMSO was used as the mobile phase at a flow rate of 1 mL/min, and the column temperature was maintained at 80 °C. PMMA standards were used for calibration.

### 3.7. Physicochemical Properties of Rice Starch

#### 3.7.1. Solubility and Swelling Power of Starch

Solubility and swelling power were determined according to Xing et al. [37] with slight modifications. Briefly, starch was dispersed in deionized water to prepare a 2% suspension and heated at 50, 70, or 90 °C for 30 min with intermittent mixing. After cooling, the suspensions were centrifuged (4000 r/min, 20 min) to separate the liquid and swollen starch fractions. The dried soluble fraction was used to determine solubility, while the hydrated sediment was used to evaluate swelling power.

#### 3.7.2. Freeze–Thaw Stability of Starch

For the freeze–thaw stability analysis, an appropriate amount of starch was mixed with distilled water to prepare a 5% starch suspension. The suspension was continuously stirred and heated at 95 °C for 30 min to achieve complete gelatinization, and was then cooled to 30 °C. Centrifuge tubes were weighed, and the mass was recorded as M_1_. Subsequently, 10.0 ± 0.5 g of the starch paste was transferred into each centrifuge tube, and the total mass was recorded as M_2_. All samples were frozen at −18 °C for 22 h and thawed at 30 °C for 2 h; this procedure was regarded as one freeze–thaw cycle. After each cycle, three tubes were randomly selected and centrifuged at 8000× *g* for 20 min. The supernatant was discarded, and the mass of the tubes containing the precipitate was weighed and recorded as M_3_. The remaining tubes were subjected to the subsequent freeze–thaw cycles. After three freeze–thaw cycles, the syneresis rate of the starch gel was determined [38] and calculated using the following formula:

(4) $$\mathrm{Syneresis}\;(\%)\;=\;\frac{M_{2}\;-\;M_{3}}{M_{2}\;-\;M_{1}}\;\times 100\%$$

where *M*_1_, *M*_2_, and *M*_3_ refer to the masses of the empty centrifuge tube, the tube with starch paste, and the tube with precipitate after centrifugation, respectively.

#### 3.7.3. Light Transmittance of Rice Starch Paste

A 1% starch suspension (*w*/*w*, d.b.) was prepared, and 5 mL was placed in a test tube. After complete gelatinization by heating, the paste was cooled to 25 °C, and its transmittance was measured at 620 nm using deionized water as the blank (A360 Ultraviolet-Visible Spectrophotometer, Aoe Instruments, Shanghai, China).

#### 3.7.4. DSC Analysis

Thermal gelatinization properties of rice starch were determined according to Yu et al. [39] with slight modifications, using a DSC25 differential scanning calorimeter (TA Instruments, New Castle, DE, USA). After achieving water balance for the starch samples (corrected by 12% wet basis), starch samples were ground and passed through a 200-mesh sieve. Then, 5 mg of starch and 10 μL of purified water were added to an aluminum pan, which was then sealed, mixed, and allowed to equilibrate at room temperature for 24 h. The samples were scanned from 30 to 95 °C at 10 °C/min, and the onset temperature (To), peak temperature (Tp), conclusion temperature (Tc), and gelatinization enthalpy (ΔH) were obtained from the DSC thermograms.

#### 3.7.5. RVA Analysis

The pasting properties of starch were determined using a rapid viscosity analyzer (RVA4500, Perten company, Stockholm, Sweden), following the method of Chen et al. [40] with slight modifications. Briefly, 3.00 g of starch (corrected at 12% moisture basis) was mixed with 25.00 g of distilled water in the RVA canister and dispersed by paddle stirring for 30 s. The slurry was subjected to a programmed heating–cooling cycle: holding at 50 °C for 1 min, heating to 95 °C at 12 °C/min, maintaining at 95 °C for 2.5 min, cooling to 50 °C at the same rate, and holding for 1.5 min. The whole test lasted 12.5 min. Paddle speed was set at 960 r/min for the initial 10 s and then maintained at 160 r/min. Pasting parameters were obtained using the instrument software.

#### 3.7.6. Rheological Properties of Rice Starch

##### Static Rheological Measurement

Steady-shear behavior of fully gelatinized starch paste was determined according to Xiong et al. [41] with slight modifications, using a Haake Mars 60 rheometer (Thermo Fisher Scientific, Waltham, MA, USA). A 2 mL portion of starch paste was loaded onto the rheometer platform and protected with silicone oil to reduce water loss. After equilibration at 25 °C for 2 min, measurements were carried out with a 35 mm aluminum parallel-plate geometry at a gap of 1 mm. The shear rate was varied from 0.01 to 100 s^−1^ at 25 °C, the scanning frequency was 1 Hz, and the resulting flow curves were analyzed using the Power Law model.

##### Dynamic Rheological Measurement

The dynamic viscoelastic properties of starch pastes were determined according to Xiong et al. [41] with slight modifications. Measurements were conducted at 25 °C using an oscillatory mode. A strain sweep was first carried out to select an appropriate strain (the range of strain was from 0.01% to 100%) within the non-destructive deformation region. Subsequently, frequency scanning was performed from 1 to 100 rad/s at the selected strain. The storage modulus (G′), loss modulus (G″), and loss tangent (tan δ) were obtained to evaluate the elastic and viscous characteristics of the starch paste system.

### 3.8. In Vitro Digestion Assay

The starch samples were subjected to gelatinization treatment (6% starch solution, boiling water bath for 30 min). The gelatinized samples were freeze-dried and then ground and dispersed, and passed through a 100-mesh sieve. To a clean 50 mL centrifuge tube, approximately 100 mg of the sample was added and dispersed with 2 mL of deionized water. Then, 8 mL of sodium acetate buffer solution (0.2 M, pH 6.0) containing 0.67 mg trypsin and 33.3 μL glucosidase was added. The mixture was mixed in a 37 °C water bath at a moderate speed of 300 rpm for incubation. Samples (0.1 mL) were taken at 0, 5, 10, 15, 20, 30, 45, 60, 90, 120, 180, 240 and 300 min and mixed with 0.9 mL of anhydrous ethanol. A 0.05 mL volume of the above liquid was transferred to a new EP tube, 1.5 mL of GOPOD reagent was added; the mixture was vortex mixed and then incubated at 50 °C for 20 min. A 0.05 mL volume of the glucose standard solution and 1.5 mL of GOPOD reagent were react at 50 °C for 20 min. The absorbance was measured at 510 nm (Multiskan GO microplate reader, Thermo Fisher Scientific, Waltham, MA, USA).

The contents of rapidly digestible starch (RDS), slowly digestible starch (SDS) and resistant starch (RS) were calculated using the following formula:

(5) $$RDS(\%)=\frac{(G_{20}-G_{0})\times 90}{TS}$$

(6) $$SDS(\%)=\frac{(G_{120}-G_{20})\times 90}{TS}$$

(7) RS(%)=100−RDS−SDS

where $G_{0}$, $G_{20}$ and $G_{120}$ represent the amounts of glucose produced 0, 20 and 120 min after the start of starch digestion, respectively; 90 is the conversion factor; and TS represents the total starch content in the sample.

### 3.9. Data Processing and Statistical Analysis

For each rice variety, starch isolated from a single grain batch was used for subsequent analyses. Unless otherwise specified, measurements were performed in triplicate using separate aliquots from the same starch preparation; these were considered technical/analytical replicates (*n* = 3). The results are expressed as mean ± standard deviation (SD) to describe analytical variability. GPC analysis was performed as a single determination for each starch sample (*n* = 1) and was therefore presented descriptively without SD or statistical significance testing.

Data processing and figure preparation were performed using OriginPro 2021 (OriginLab Corporation, Northampton, MA, USA), and statistical analyses were conducted using IBM SPSS Statistics 26.0 (IBM Corporation, Armonk, NY, USA). For measurements obtained under a single experimental condition, differences among the six rice varieties were evaluated by one-way analysis of variance (ANOVA) followed by Duncan’s multiple range test at *p* < 0.05. For solubility and swelling-power measurements conducted at 50, 70, and 90 °C, varietal comparisons were performed separately at each temperature. For freeze–thaw stability, three separate sample tubes were analyzed at each freeze–thaw cycle, and varietal comparisons were conducted separately within each cycle. Rheological shear-rate and frequency-sweep data were treated as continuous response profiles, and individual points within each sweep were not considered independent replicates. Exploratory correlation analysis was performed using the variety-level mean values of the six rice varieties (*n* = 6) to examine associations among selected compositional, structural, physicochemical, and in vitro digestibility parameters.

## 4. Conclusions

This study systematically compared the chemical composition, multiscale starch structure, physicochemical characteristics, rheological properties, and in vitro digestibility of six selected stress-resistant rice varieties. Marked varietal differences were observed in starch, amylose, protein, and mineral contents, as well as in starch granule morphology, particle size distribution, relative crystallinity, short-range molecular order, lamellar organization, and apparent molecular weight distribution. Although all isolated starches exhibited typical A-type crystalline patterns, differences in their structural characteristics were accompanied by variations in swelling behavior, freeze–thaw stability, paste clarity, thermal transitions, pasting profiles, rheological properties, and in vitro digestibility. Jingliangyou 3261 showed the highest RS content, whereas Xinjiang aromatic rice exhibited the highest SDS and lowest RDS contents. Ningjing 48 and Xinjiang aromatic rice also showed comparatively better freeze–thaw stability, and Xinjiang aromatic rice had the highest paste clarity. These findings provide comparative information on the starch quality of the selected varieties and may assist in identifying candidates for further food-processing and nutritional evaluation. However, because the present study was conducted using isolated starch rather than cooked rice or processed rice products, the potential applications of these varieties should be further validated under practical cooking and food-processing conditions.

## Figures and Tables

**Table 1:** Chemical composition of rice flours from the six selected rice varieties.

Variety	Moisture (%)	Total Starch (%)	Amylose (%)	Protein (%)	Crude Fat (%)	Ash (%)	Crude Fiber (%)
Shaoxiang 100	12.46 ± 0.04 ^d^	78.34 ± 3.26 ^c^	15.35 ± 0.07 ^c^	5.23 ± 0.10 ^d^	0.85 ± 0.01 ^a^	0.54 ± 0.01 ^a^	1.3 ± 0.2 ^a^
Jingliangyou 3261	13.63 ± 0.07 ^b^	83.51 ± 3.01 ^a^	12.67 ± 0.13 ^f^	6.05 ± 0.14 ^c^	0.73 ± 0.01 ^b^	0.54 ± 0.01 ^a^	1.4 ± 0.5 ^a^
Ningjing 48	12.89 ± 0.07 ^c^	80.69 ± 1.09 ^b^	18.12 ± 0.11 ^a^	5.99 ± 0.20 ^c^	0.47 ± 0.02 ^d^	0.35 ± 0.01 ^c^	1.2 ± 0.1 ^b^
Xinjiang aromatic rice	12.27 ± 0.01 ^e^	83.14 ± 0.80 ^a^	16.94 ± 0.13 ^b^	6.88 ± 0.10 ^b^	0.37 ± 0.01 ^e^	0.30 ± 0.04 ^d^	1.2 ± 0.3 ^bc^
Xizi 3	13.60 ± 0.01 ^b^	79.34 ± 2.04 ^bc^	15.81 ± 0.04 ^c^	7.50 ± 0.24 ^a^	0.43 ± 0.02 ^de^	0.27 ± 0.01 ^d^	1.1 ± 0.1 ^cd^
Zhenliangyou 8612	15.90 ± 0.10 ^a^	78.11 ± 0.36 ^c^	13.71 ± 0.04 ^e^	6.42 ± 0.26 ^bc^	0.53 ± 0.04 ^c^	0.48 ± 0.04 ^b^	1.1 ± 0.1 ^d^

Note: Values are expressed as mean ± SD, *n* = 3. Different lowercase letters within the same column indicate significant differences among samples at *p* < 0.05.

**Table 2:** Particle size parameter distribution of starches from different rice varieties.

Variety	D _[4,3]_	D _[3,2]_	D_(10)_	D_(50)_	D_(90)_
Shaoxiang 100	0.703 ± 0.000 ^c^	0.692 ± 0.001 ^e^	0.597 ± 0.001 ^d^	0.699 ± 0.000 ^c^	0.817 ± 0.000 ^b^
Jingliangyou 3261	0.704 ± 0.000 ^b^	0.694 ± 0.000 ^b^	0.599 ± 0.000 ^b^	0.700 ± 0.000 ^b^	0.817 ± 0.000 ^b^
Ningjing 48	0.692 ± 0.001 ^d^	0.682 ± 0.000 ^d^	0.591 ± 0.001 ^e^	0.686 ± 0.000 ^e^	0.806 ± 0.000 ^c^
Xinjiang aromatic rice	1.381 ± 0.000 ^a^	1.372 ± 0.000 ^a^	1.246 ± 0.001 ^a^	1.376 ± 0.000 ^a^	1.526 ± 0.000 ^a^
Xizi 3	0.703 ± 0.000 ^c^	0.693 ± 0.000 ^c^	0.598 ± 0.000 ^c^	0.698 ± 0.001 ^d^	0.817 ± 0.001 ^b^
Zhenliangyou 8612	0.704 ± 0.000 ^b^	0.693 ± 0.000 ^c^	0.599 ± 0.000 ^b^	0.699 ± 0.000 ^c^	0.817 ± 0.000 ^b^

Note: Values are expressed as mean ± SD, *n* = 3. Different lowercase letters within the same column indicate significant differences among samples at *p* < 0.05. D _[4,3]_ represents the De Brouckere mean diameter, and D _[3,2]_ represents the Sauter mean diameter. D_(10)_, D_(50)_ and D_(90)_ represent the particle sizes corresponding to the cumulative distribution percentages of 10%, 50% and 90%, respectively.

**Table 3:** Peak position and lamellar repeat distance of starches from different stress-resistant rice varieties.

Variety	q_Peak (Å^−1^)	d (nm)
Shaoxiang 100	0.0710 ± 0.0001	8.85 ± 0.01 ^e^
Jingliangyou 3261	0.0704 ± 0.0002	8.93 ± 0.02 ^d^
Ningjing 48	0.0671 ± 0.0002	9.36 ± 0.03 ^a^
Xinjiang aromatic rice	0.0683 ± 0.0002	9.20 ± 0.02 ^b^
Xizi 3	0.0689 ± 0.0002	9.12 ± 0.02 ^c^
Zhenliangyou 8612	0.0711 ± 0.0001	8.84 ± 0.01 ^e^

Note: Values are expressed as mean ± SD (*n* = 3). Different lowercase letters within the same column indicate significant differences among rice varieties (*p* < 0.05).

**Table 4:** Apparent molecular-weight distribution of starches from different rice varieties.

Variety	Mp	Mn	Mw	Mz	Mz + 1	Mv	PDI
Shaoxiang 100	7.27 × 10^6^	3.04 × 10^5^	4.93 × 10^6^	1.04 × 10^8^	3.07 × 10^8^	2.65 × 10^6^	16.2167
Jingliangyou 3261	1.03 × 10^7^	4.18 × 10^5^	8.62 × 10^6^	1.17 × 10^8^	2.76 × 10^8^	4.83 × 10^6^	20.6316
Ningjing 48	8.34 × 10^6^	4.13 × 10^5^	9.10 × 10^6^	1.76 × 10^8^	4.95 × 10^8^	4.87 × 10^6^	22.0306
Xinjiang aromatic rice	1.28 × 10^7^	3.90 × 10^5^	1.28 × 10^7^	1.92 × 10^8^	3.95 × 10^8^	6.66 × 10^6^	32.8234
Xizi 3	7.65 × 10^6^	3.63 × 10^5^	9.97 × 10^6^	2.15 × 10^8^	4.89 × 10^8^	4.88 × 10^6^	27.4376
Zhenliangyou 8612	8.95 × 10^6^	3.56 × 10^5^	1.03 × 10^7^	1.88 × 10^8^	4.14 × 10^8^	5.25 × 10^6^	28.9593

Note: Mp (peak molecular weight); Mn (number average molecular weight); Mw (weight average molecular weight); Mz (z average molecular weight); Mz + 1 (z + 1 average molecular weight); Mv (viscosity average molecular weight); PDI (polydispersity index).

**Table 5:** In vitro digestibility characteristics of starches from different rice varieties.

Variety	RDS (%)	SDS (%)	RS (%)
Shaoxiang 100	64.14 ± 0.93 ^c^	31.65 ± 0.80 ^b^	4.46 ± 0.47 ^c^
Jingliangyou 3261	60.54 ± 0.77 ^d^	28.65 ± 0.73 ^c^	10.59 ± 0.43 ^a^
Ningjing 48	72.74 ± 0.78 ^a^	22.45 ± 0.70 ^e^	4.68 ± 0.52 ^c^
Xinjiang aromatic rice	60.44 ± 0.77 ^d^	33.75 ± 0.91 ^a^	5.57 ± 0.46 ^c^
Xizi 3	72.79 ± 0.82 ^a^	22.40 ± 0.59 ^e^	4.79 ± 0.54 ^c^
Zhenliangyou 8612	66.63 ± 0.83 ^b^	24.69 ± 0.73 ^d^	8.62 ± 0.47 ^b^

Note: Values are expressed as mean ± SD. Different lowercase letters within the same column indicate significant differences among varieties (*p* < 0.05). RDS (rapidly digestible starch), SDS (slowly digestible starch), and RS (resistant starch).

## Data Availability

Dataset available on request from the authors.

## Supplementary Materials

The following supporting information can be downloaded at: https://www.mdpi.com/article/10.3390/molecules31183255/s1, Table S1: Trace element concentrations in stress-resistant rice grains; Table S2: Solubility and swelling power of rice starches at different temperatures; Table S3: Thermal properties of rice starches determined by DSC; Figure S1: Pearson correlation analysis.
