# Supplementary material for: Comparative Characterization of the Multiscale Structure, Physicochemical Properties, and In Vitro Digestibility of Starches from Selected Stress-Resistant Rice Varieties
Source: Molecules. 2026 Sep 14;31(18):3255. doi: 10.3390/molecules31183255 (PMC13609739; doi:10.3390/molecules31183255)
Supplement: Supplementary file 1 [file molecules-31-03255-s001.zip › molecules-4517074-supplementary.pdf]

Table S1. Trace element concentrations in stress-resistant rice grains

Variety	Fe	Zn	Mg	Ca	Mn	Cu	Cd	Se
Shaoxiang 100	32.82 ± 1.12^{b}	16.28 ± 0.43^{b}	418.75 ± 12.46^{a}	68.32 ± 2.05^{a}	4.64 ± 0.18^{d}	2.32 ± 0.09^{bc}	N.D.	0.04 ± 0.01^{c}
Jingliangyou 3261	89.88 ± 2.67^{a}	18.79 ± 0.51^{a}	356.37 ± 10.82^{b}	67.53 ± 1.88^{a}	10.32 ± 0.32^{a}	3.46 ± 0.13^{a}	N.D.	N.D.
Ningjing 48	25.83 ± 0.86^{c}	14.48 ± 0.38^{c}	70.28 ± 2.31^{e}	55.25 ± 1.63^{b}	6.25 ± 0.21^{c}	2.14 ± 0.08^{c}	N.D.	0.09 ± 0.01^{b}
Xinjiang aromatic rice	24.11 ± 0.79^{cd}	15.52 ± 0.42^{bc}	148.81 ± 4.76^{c}	52.65 ± 1.57^{b}	4.85 ± 0.17^{d}	1.77 ± 0.07^{d}	N.D.	0.09 ± 0.01^{b}
Xizi 3	21.95 ± 0.72^{d}	13.58 ± 0.36^{d}	98.52 ± 3.28^{d}	34.82 ± 1.08^{c}	7.99 ± 0.26^{b}	3.58 ± 0.14^{a}	0.03 ± 0.01^{a}	0.05 ± 0.01^{c}
Zhenliangyou 8612	23.47 ± 0.81^{cd}	11.00 ± 0.31^{e}	137.05 ± 4.35^{c}	35.96 ± 1.12^{c}	3.37 ± 0.13^{e}	2.49 ± 0.10^{b}	0.04 ± 0.01^{a}	0.47 ± 0.02^{a}

Note: Values are expressed as mean ± SD, n = 3. Different lowercase letters within the same column indicate significant differences among samples at $p < 0.05$. N.D., not detected. Cadmium detection limit: 0.002 mg/kg, Selenium detection limit: 0.01 mg/kg. All element concentrations are expressed as mg kg^{-1}.

Table S2. Solubility and swelling power of rice starches at different temperatures

**Sample**	**Solubility (%)**			**Swelling power (g/g)**		
	**50°C**	**70°C**	**90°C**	**50°C**	**70°C**	**90°C**
Shaoxiang 100	1.21 ± 0.18[b]	3.76 ± 0.11[c]	10.04 ± 0.16[c]	2.37 ± 0.14[c]	9.85 ± 0.22[a]	17.55 ± 0.13[a]
Jingliangyou 3261	1.18 ± 0.06[b]	2.03 ± 0.23[d]	6.46 ± 0.09[d]	2.42 ± 0.10[bc]	4.46 ± 0.18[d]	14.56 ± 0.09[c]
Ningjing 48	2.25 ± 0.15[a]	5.08 ± 0.10[a]	12.18 ± 0.28[b]	2.45 ± 0.16[bc]	8.50 ± 0.11[b]	16.03 ± 0.27[b]
Xinjiang aromatic rice	1.36 ± 0.09[b]	4.92 ± 0.27[a]	12.54 ± 0.12[b]	2.70 ± 0.08[a]	8.97 ± 0.24[ab]	16.50 ± 0.15[ab]
Xizi 3	1.02 ± 0.21[b]	2.81 ± 0.07[d]	10.36 ± 0.18[c]	2.55 ± 0.12[ab]	6.92 ± 0.19[c]	14.91 ± 0.10[c]
Zhenliangyou 8612	1.20 ± 0.12[b]	3.62 ± 0.31[c]	14.94 ± 0.22[a]	2.56 ± 0.18[ab]	3.18 ± 0.07[e]	16.86 ± 0.23[ab]

Note: Values are expressed as mean ± SD. Different lowercase letters within the same column indicate significant differences among varieties at the same temperature ($p < 0.05$).

Table S3. Thermal properties of rice starches determined by DSC

**Variety**	**To (°C)**	**Tp (°C)**	**Tc (°C)**	**ΔH (J/g)**
Shaoxiang 100	64.65 ± 0.21	70.35 ± 0.14	76.63 ± 0.16	11.12 ± 0.70
Jingliangyou 3261	75.60 ± 0.18	81.60 ± 0.04	86.48 ± 0.22	12.03 ± 0.66
Ningjing 48	60.16 ± 0.20	66.72 ± 0.02	75.80 ± 0.14	9.79 ± 0.52
Xinjiang aromatic rice	59.90 ± 0.29	66.38 ± 0.03	75.57 ± 0.54	8.65 ± 0.21
Xizi 3	66.52 ± 0.28	72.78 ± 0.02	79.56 ± 0.31	12.21 ± 0.13
Zhenliangyou 8612	75.39 ± 0.18	81.14 ± 0.21	87.33 ± 0.65	14.93 ± 0.75

Note: Values are expressed as mean ± SD (n = 3). To, onset temperature; Tp, peak temperature; Tc, conclusion temperature; ΔH, gelatinization enthalpy.

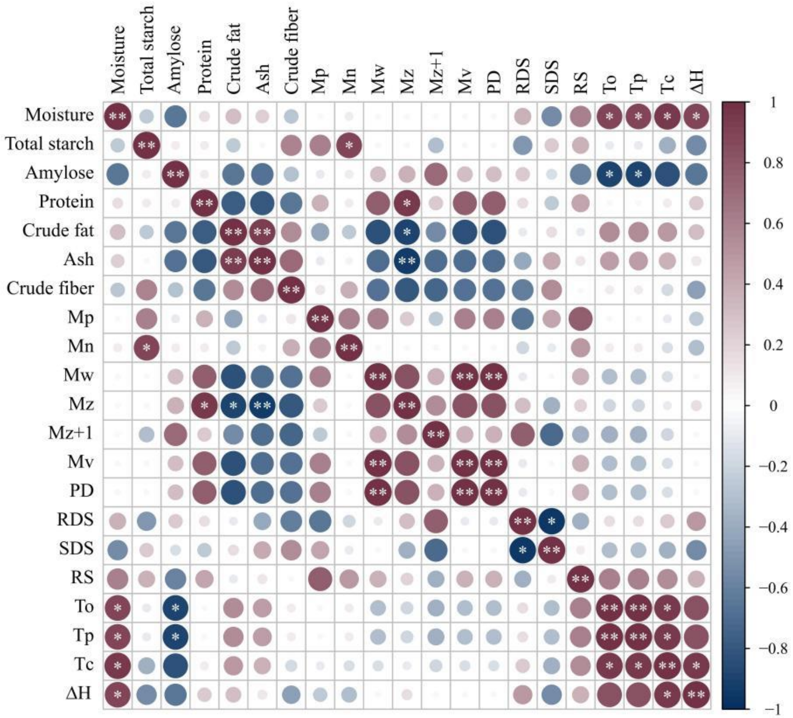

Figure S1. Pearson correlation analysis among the chemical composition, molecular-weight characteristics, thermal properties, and *in vitro* digestibility of starches from six stress-resistant rice varieties. Red and blue circles represent positive and negative correlations, respectively. The size and color intensity of the circles indicate the magnitude of the correlation coefficients. *: $p < 0.05$, **: $p < 0.01$.
